# Transcriptional profiles in the chicken ductus arteriosus during hatching

**DOI:** 10.1371/journal.pone.0214139

**Published:** 2019-03-21

**Authors:** Toru Akaike, Satoko Shinjo, Eriko Ohmori, Ichige Kajimura, Nobuhito Goda, Susumu Minamisawa

**Affiliations:** 1 Department of Cell Physiology, The Jikei University School of Medicine, Tokyo, Japan; 2 Department of Life Science and Medical Bioscience, Waseda University, Tokyo, Japan; Chuo University, JAPAN

## Abstract

The ductus arteriosus, an essential embryonic blood vessel between the pulmonary artery and the descending aorta, constricts after birth or hatching and eventually closes to terminate embryonic circulation. Chicken embryos have two long ductus arteriosi, which anatomically differ from mammal ductus arteriosus. Each long ductus arteriosus is divided into two parts: the pulmonary artery-sided and descending aorta-sided ductus arteriosi. Although the pulmonary artery-sided and descending aorta-sided ductus arteriosi have distinct functional characteristics, such as oxygen responsiveness, the difference in their transcriptional profiles has not been investigated. We performed a DNA microarray analysis (GSE 120116 at NCBI GEO) with pooled tissues from the chicken pulmonary artery-sided ductus arteriosus, descending aorta-sided ductus arteriosus, and aorta at the internal pipping stage. Although several known ductus arteriosus-dominant genes such as *tfap2b* were highly expressed in the pulmonary artery-sided ductus arteriosus, we newly found genes that were dominantly expressed in the chicken pulmonary artery-sided ductus arteriosus. Interestingly, cluster analysis showed that the expression pattern of the pulmonary artery-sided ductus arteriosus was closer to that of the descending aorta-sided ductus arteriosus than that of the aorta, whereas the morphology of the descending aorta-sided ductus arteriosus was closer to that of the aorta than that of the pulmonary artery-sided ductus arteriosus. Subsequent pathway analysis with DAVID bioinformatics resources revealed that the pulmonary artery-sided ductus arteriosus showed enhanced expression of the genes involved in melanogenesis and tyrosine metabolism compared with the descending aorta-sided ductus arteriosus, suggesting that tyrosinase and the related genes play an important role in the proper differentiation of neural crest-derived cells during vascular remodeling in the ductus arteriosus. In conclusion, the transcription profiles of the chicken ductus arteriosus provide new insights for investigating the mechanism of ductus arteriosus closure.

## Introduction

The ductus arteriosus (DA), an essential embryonic blood vessel between the pulmonary artery (PA) and the descending aorta, exists in all terrestrial vertebrates. The DA works as a right-to-left shunt in these animals to run the oxygenated blood from the embryonic gas exchanger directly into the descending aorta. Mammals use the placenta from maternal circulation for the embryonic gas exchanger system, whereas oviparous animals such as birds and reptiles utilize the chorioallantoic membrane. Despite the differences in the gas exchanger systems, the DA shares similar physiological functions in all terrestrial vertebrates. For example, the DA constricts after birth or hatching due to an increase in the oxygen tension associated with the first breath, and it eventually closes to terminate embryonic circulation. [1, 2].

In addition to the differences in the embryonic gas exchanger systems, the anatomy of the DA in oviparous animals is different from that in mammals. Most studies on oviparous ductus arteriosi have been conducted using chick embryos. Chicks have two, long ductus arteriosi that branch off from the right and left pulmonary arteries, whereas mammals have a single, short DA [3]. Both the right and left ductus arteriosi in chicks exhibit two distinct parts: the pulmonary artery-sided (proximal) and descending aorta-sided (distal) ductus arteriosi. The proximal DA, from the orifice of the pulmonary arteries to its middle portion, exhibits a muscular type of artery [4–6]. The distal DA, from the middle portion to the orifice of the descending aorta, exhibits an elastic type of artery that is similar to the aorta [4–6]. It has been shown that the proximal DA is completely closed by 2 days after hatching, whereas the distal DA remains open even after hatching [7]. In addition, the functional character between the proximal and distal ductus arteriosi is known to differ; for example, the proximal DA, but not the distal DA constricts in response to oxygen [1, 2]. Therefore, the proximal DA in oviparous animals is thought to be comparable to the mammalian DA.

To date, the characteristic features of DA closure are known to occur by a combination of two events: functional constriction and morphological remodeling in all terrestrial vertebrates. These characteristics are based on the specific transcriptional profiles of the DA. Several studies, including ours, have identified a unique transcriptional profile of the mammalian DA in humans [8, 9], baboons [10], lambs [11], and rodents [12–18] via DNA microarray analyses or RNA sequence analysis. However, no study has yet been conducted to examine a transcriptional profile of the DA of oviparous animals such as chicks. Because the mechanisms of DA closure are thought to share common processes between mammalian and oviparous animals, it is of interest to compare the transcriptional profiles of the chicken DA with those of mammals.

## Materials and methods

### Animals

Fertilized Hypeco Nera chickens (Gallus gallus domesticus) were obtained from Shiroyama Keien Farms (Kanagawa, Japan). Eggs that were incubated at 37.5°C at a relative humidity of 60–75% were turned every hour. The DA and its connecting arteries including the pulmonary arteries and the descending aorta from five different ages of developing chickens were isolated: prepipped embryonic day 16 (ED16), day 19 (ED19), internal pipping (IP), external pipping (EP), and one day after birth (AB), which were used within 24 hours of hatching. At the IP stage, embryos break into the air cell with their beaks and still breathe hypoxic air, but after the EP phase, embryos break the eggshell and start to breathe normoxic air. Animals were euthanized with isoflurane, and all efforts were made to minimize suffering. The protocol of animal experiments was reviewed and approved by the Institutional Animal Care and Use Committee of The Jikei University (No. 2015–143) and conformed to the Guidelines for the Proper Conduct of Animal Experiments of the Science Council of Japan (2006).

### Histological analyses

In order to histologically analyze the chicken DA at ED16, ED19, IP, EP, and AB with an optical microscope BZ-9000 (KEYENCE Inc.), they were fixed with 10% neutral buffered formalin fixative, and embedded with paraffin. Finally, the 4-μm serial paraffin sections were stained with hematoxylin-eosin (HE) staining and Elastica van Gieson (EVG) staining. The vessel wall thickness was measured at four different parts in each vessel by using the ImageJ 1.51 program, and results were obtained from the average of four parts in each vessel. The vessel luminal area was measured in each vessel by using ImageJ 1.51 program.

### DNA microarray

Pooled tissues from ten embryos of the proximal and distal DA of the right-sided DA and the descending aorta at the IP stage were collected separately for each sample of RNA isolation. Sepasol-RNA I Super G (Nacalai Tesque, Inc., Japan) was used as recommended by the manufacturer. RNA purity of the samples was confirmed to have an OD260/280 ratio between 1.8 and 2.0 by UV spectrophotometry. Total RNA was converted to cDNA using Ambion Whole Transcript Expression Kit for Affymetrix GeneChip Whole Transcript Expression Arrays. cDNA was then biotin-labeled and hybridized to a GeneChip Chicken Gene 1.0 ST Array (Affymetrix, Santa Clara, CA, USA). Briefly, a total of 100 ng of total RNA was reverse-transcribed to cDNA, which was subsequently used as a template for an *in vitro* transcription reaction. Sense-strand cDNA containing dUTP was synthesized by amplified cRNA. The Affymetrix GeneChip Whole Transcript Terminal Labeling Kit (Affymetrix, Santa Clara, CA, USA) was used to recognize the dUTP and to fragment the cDNA with uracil-DNA glycosylase and apurinic/apyrimidinic endonuclease 1. These fragmented cDNAs were then labeled through a terminal deoxy-transferase reaction and hybridized to the Affymetrix GeneChip Chicken Gene 1.0 ST-v1 Array (Affymetrix). The arrays were incubated in a 45°C hybridization oven, at 60 rpm, for 17 hours. After incubation, the arrays were washed, stained, and scanned using an Affymetrix GeneChip Scanner. The data were analyzed with Affymetrix GeneChip Command Console Software version 2.0 and then exported as Affymetrix CEL files. Normalization of the raw data, quality control checks, summarized to probeset expression estimates through robust multi-array average (RMA) were performed using the oligo package (v1.40.1) in Bioconductor (v3.5). The hybridization experiments were performed in duplicate, and the intensities in the proximal DA, the distal DA, and the descending aorta were averaged.

### Hierarchical clustering analysis

The similarity matrix of normalized data described above was computed using the Dist function from amap package (v0.8–14) using centered Pearson’s correlation coefficient and clustered using the hclust function from stats package (v3.5.0) using Ward’s method for agglomeration.

### Gene ontology analysis

We took the top 0.5% (88 genes) in the ratio of signal intensities of the proximal DA to the distal DA and asked which GO terms in the KEGG pathway were enriched in the genes using DAVID knowledgebase v6.8 [19]. Only pathways with a *p*-value lower than 0.05 were considered to be significantly enriched.

### RNA extraction and real-time polymerase chain reaction

Pooled tissues from the proximal and distal DA of the right pulmonary artery side and the descending aorta were collected separately at five different developmental stages: ED16, ED19, IP, EP, and AB. Total RNA was extracted from these pooled tissues using Sepasol-RNA I Super G (Nacalai tesque, Inc., Japan) and then reverse-transcribed to cDNA using a PrimeScript™ RT Master Mix (TAKARA bio Inc., Japan) as recommended by the manufacturer. cDNA was amplified using TB Green™ Premix Ex Taq II for intercalator-based quantitative polymerase chain reaction (qPCR), or Probe qPCR Mix for TaqMan probe-based qPCR (Takara Bio Inc., Japan). Sequences for PCR primers and probes are listed in Table 1. Duplicate reactions were performed for each sample. The reactions were performed in 96-well plates on the Thermal Cycler Dice Real Time System (Takara Bio Inc., Japan). For each RT-PCR experiment, a negative control was included, and no amplification was confirmed in any of the reactions. For data analysis, the mRNA levels of interest were normalized to chicken *gapdh* mRNA.

**Table 1:** Primer and probe sequences.

Gene name	Forward primer	Reverse primer	Probe
*col8a1*	GCGACTGAGAACAGAGTAGGTCAG	AAACCCCACCAACCACCA	
*tnc*	GAGACGGCACAACTTCTCTG	CCTTTCCTTTCTTGGCACAC	
*mamdc2*	ACTGGAGACTGGGTGTTAGCTG	AGGGCAACATAACCTCCCTTGG	
*hpse2*	TGCTCTGCGTCGAAATCCCA	TGAGCGACCGATCAATGTCC	
*fam19a2*	GGCACTCCACAGATGTTGCA	CCCTTCAAGACATGGTTGCATG	
*dct*	GCCCCTCAAGTTCTTCAACCAG	TTCCTGACAACTGGTGGCTTC	
*ednrb2*	TCTACAATGCTGCCATCCTCCTG	TCTGACCCTTCAGCCAGGTTC	
*tyr*	TGACAGCATTTTTGAGCGGTGG	TGATGGGCTTGCTTGAGGTAGG	CCAGCAGCCAACGCACCCAT
*tyrp1*	ATTCCACTTTCGGCCTACCC	CGTTGTACCATAGGCCGTGC	TCAGTGGCGTGTGCTGTGTGA
*wnt11*	TCAGGTTCTGGCTCATTCACAC	GGCTGTCCACATCACAATCCAATC	
*gapdh*	GACAACTTTGGCATTGTGGAG	CAGGTCAGGTCAACAACAGAG	

### Statistical analysis

**Fig 1:**
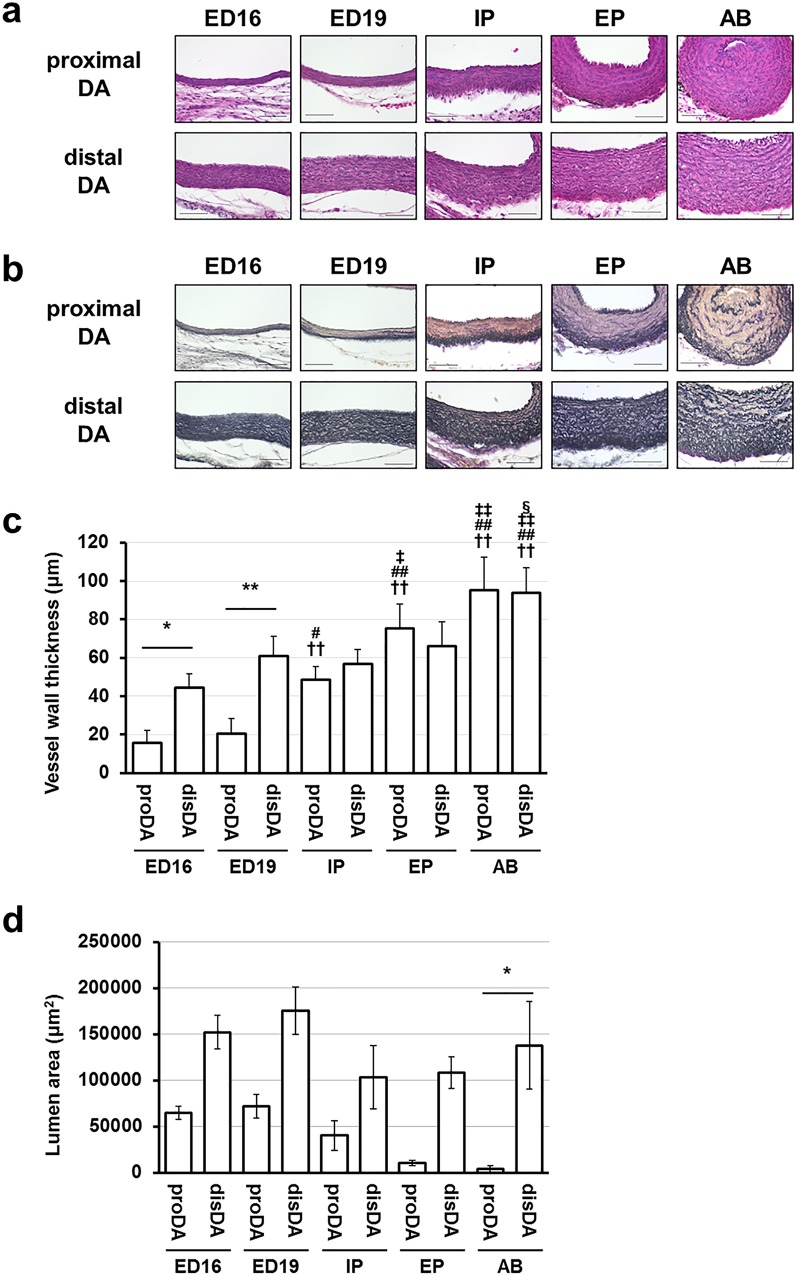
Distinct morphological changes in the proximal portions of the chick DA during development. Representative histological sections of the proximal and distal DA from ED16 to AB. Serial sections were stained with HE (a) and EVG staining (b). (a) The nucleus in the middle lamella of the distal DA were aligned in parallel to the internal elastic laminar and layered throughout the hatching, whereas the layers were disorganized in the proximal DA from the IP stage. Scale bars are 50 μm. (b) The proximal DA and the distal DA had a muscular and an elastic type of artery, respectively. Moreover, in the proximal DA, the poor elastic fiber formation was observed in the middle lamella from the IP stage. Scale bars are 50 μm. (c) The vessel wall thickness of the chick DA at developmental stages. The vessel wall of the proximal DA was thin compared to the distal DA at the ED16 and ED19 stage. Moreover, the vessel wall of the proximal DA drastically thickened from the ED19 to the IP stage. * and ** indicate *p* < 0.05 and *p*<0.01, †† indicates *p*<0.01 compared to the same tissues of the ED16 stage, # and ## indicate *p* < 0.05 and *p*<0.01 compared to the same tissues of the ED19 stage, ‡ and ‡‡ indicate *p* < 0.05 and *p*<0.01 compared to the same tissues of the IP stage, § indicates *p*<0.05 compared to the same tissues of the EP stage, n = 4–5. (d) The vessel luminal area of the chick DA at developmental stages. * indicates *p* < 0.05. n = 4–5.

**Fig 2:**
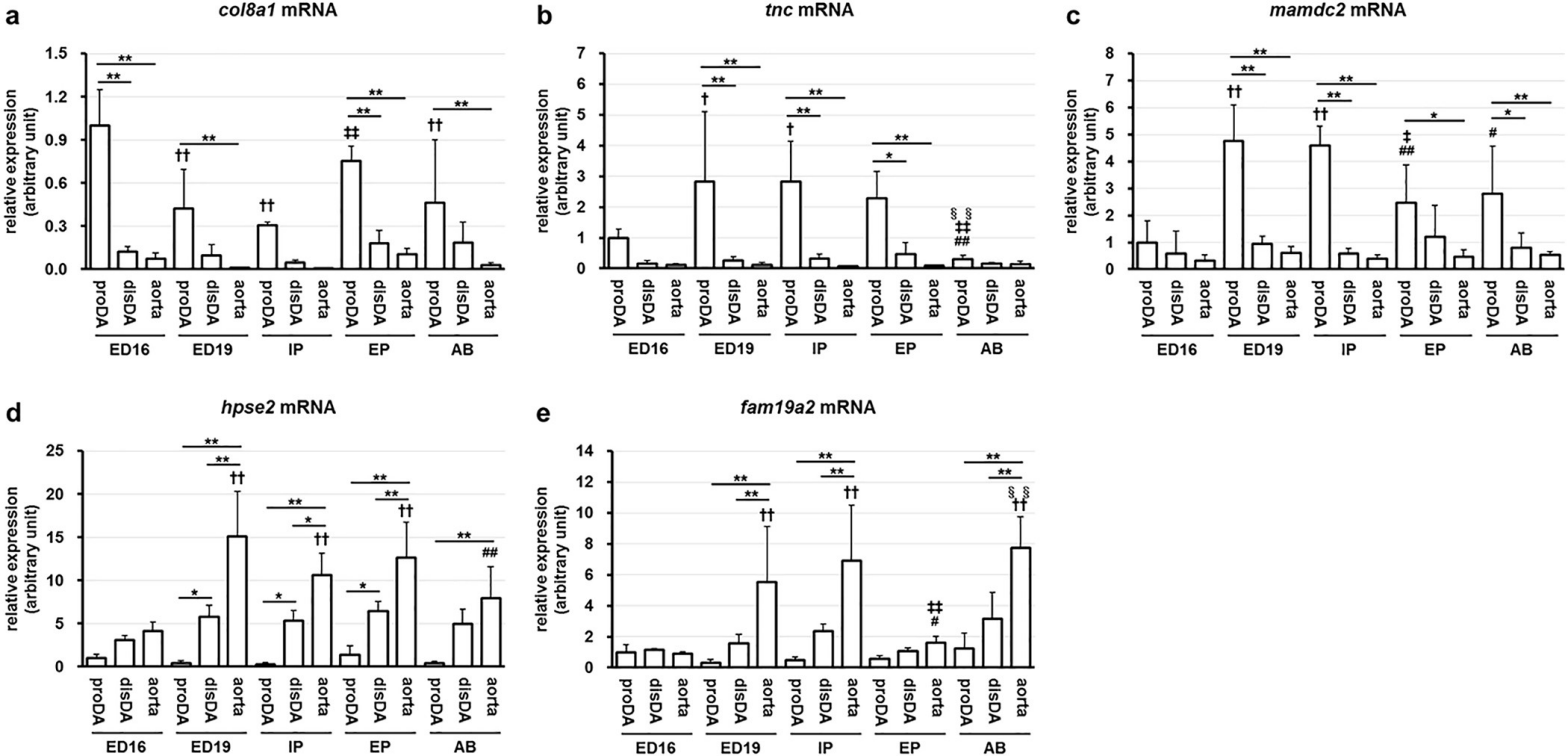
Quantitative PCR analysis for proximal DA-enriched or aorta-enriched genes. The relative mRNA levels of *col8a1* (a), *tnc* (b), *mamdc2* (c), *hpse2* (d) or *fam19a2* (e) to *gapdh* were quantified by RT-PCR. * and ** indicate *p* < 0.05 and *p*<0.01, † and †† indicate *p* < 0.05 and *p*<0.01 compared to the same tissues of the ED16 stage, # and ## indicate *p* < 0.05 and *p*<0.01 compared to the same tissues of the ED19 stage, ‡ and ‡‡ indicate *p* < 0.05 and *p*<0.01 compared to the same tissues of the IP stage, §§ indicates *p*<0.01 compared to the same tissues of the EP stage, n = 5–6.

**Fig 3:**
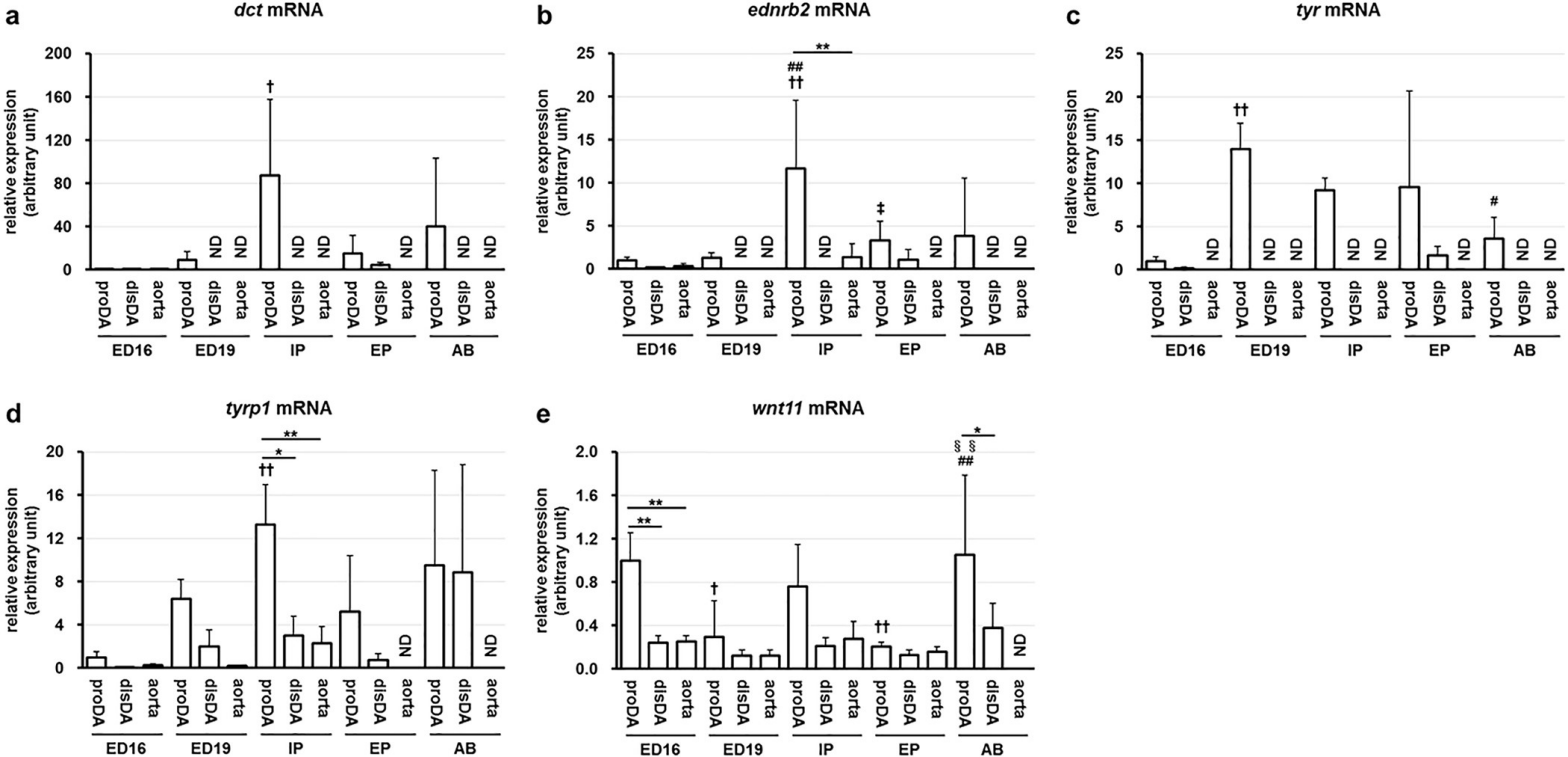
Quantitative PCR analysis for melanogenesis/tyrosine metabolism-related genes. The relative mRNA levels of each gene, *dct* (a), *ednrb2* (b), *tyr* (c), *tyrp1* (d) or *wnt11* (e), to *gapdh* were quantified by RT-PCR. We performed this experiment in quintuplicate and noted results as ND only if we could not detect any signals from two or more samples among the five available samples. * and ** indicate *p* < 0.05 and *p*<0.01, † and †† indicate *p* < 0.05 and *p*<0.01 compared to the same tissues of the ED16 stage, # and ## indicate *p* < 0.05 and *p*<0.01 compared to the same tissues of the ED19 stage, ‡ and ‡‡ indicate *p* < 0.05 and *p*<0.01 compared to the same tissues of the IP stage, §§ indicates *p*<0.01 compared to the same tissues of the EP stage, n = 5. ND: not detected.

All data are presented as the mean ± standard deviation. Statistical differences between groups were analyzed by two-way analysis of variance to evaluate the interaction between the developmental stage and the vessel type in Figs 1C, 1D and 2. If the interaction effect is present, multiple tests are being performed by a Tukey test for *post hoc* comparisons using GraphPad Prism version 8.0.1 for Windows (GraphPad Software, San Diego California USA). Statistical differences between groups are not being performed by two-way analysis of variance in Fig 3 because the expression of some groups was below the detection limit. Instead, statistical differences between groups were analyzed by one-way analysis of variance in Fig 3, and multiple tests are being performed by a Tukey test for *post hoc* comparisons using GraphPad Prism version 8.0.1 for Windows. Statistical differences were considered significant at *p*-values < 0.05.

## Results

### Histological analyses revealed distinct morphological changes in the proximal portions of the chick DA during development

As described in the introduction, the right and left ductus arteriosi in chicks exhibit two distinct parts: the proximal and distal ductus arteriosi that are separated in their middle portions. The morphology of the right and left chicken ductus arteriosi is known to be very similar during development [7], and we also confirmed this similarity (data not shown). Several groups studying chicken DA have also used the right DA [1, 2], so we also used the right DA in the present study.

The wall thickness of the proximal DA was significantly thinner than that of the distal DA at ED16 and ED19 (Fig 1A and 1C). At ED19, part of the wall of the proximal DA became thicker, making its wall thickness uneven. The tunica media of the proximal DA exhibited rapid development after ED19. The vessel wall thickness was significantly increased at the IP stage when compared with that at ED19 (Fig 1C), although the luminal cross sectional area did not significantly changed from ED19 to the IP stage (Fig 1D). On the other hand, the wall thickness of the distal DA showed small increases at the ED16, ED19, and IP stages. Therefore, there was no significant difference in the wall thickness between the proximal and distal ductus arteriosi at the IP stage and after that (Fig 1C). In addition to the wall thickness, the apparent difference between the proximal and distal portions was elastic fiber formation in the tunica media (Fig 1B). In the proximal DA, the elastic tissue was sparse in smooth muscle cells in all stages examined. In contrast, the elastic fibers were well developed in all medial walls of the distal DA as “elastic vessels” before hatching. At one day after birth (AB), the lumen of the proximal DA was almost closed, whereas that of the distal DA remained widely open.

### DNA microarray analyses revealed distinct transcriptional profiles of the chick DA at IP

**Fig 4:**
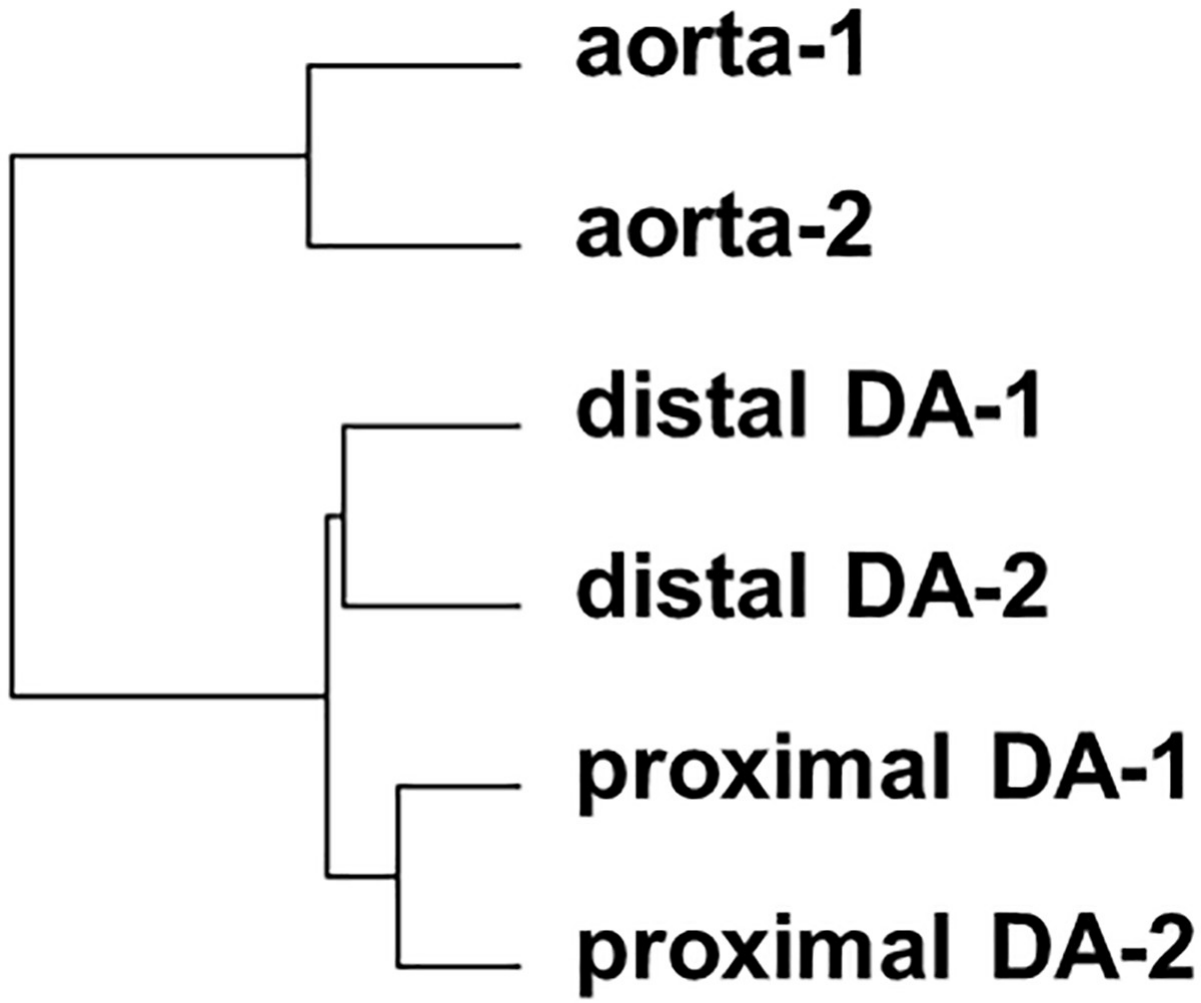
Hierarchical clustering of normalized DNA array data. The graphical representation showed that the profiles of gene expression were grouped into two distinct clusters: a DA-related cluster and an aorta-related cluster (distance = 1 − Pearson’s correlation coefficient, Ward’s method).

We performed microarray analysis with the proximal and distal DA of the right-sided DA at the IP stage. The degree of similarity of the transcriptional profiles of the proximal DA, distal DA, and the descending aorta at IP was calculated, and the graphical representation showed that the profiles of gene expression are grouped into two distinct clusters: a DA-related cluster and an aorta-related cluster (Fig 4). Although the histological analysis indicated that the distal DA was more similar to the aorta than the proximal DA, the transcriptional profiles of the proximal DA and distal DA were much closer than that of the aorta. The top 30 genes for which expression levels were higher in the proximal DA or the distal DA than in the aorta are shown in S1 Table or S2 Table. Among these 30 genes, 17 genes such as tenascin C are highly expressed in both the proximal and distal DA. The top 30 genes for which expression levels were lower in the proximal DA or the distal DA than in the aorta are shown in S3 Table or S4 Table. Among these 30 genes, 15 genes such as microRNA mir-1329 are more highly expressed in the descending aorta than in both the proximal and distal DA.

Because it has been known that, in addition to the structural difference, the proximal and distal ductus arteriosi have distinct functional characteristics such as oxygen responsiveness, the difference of the transcriptional profiles should be of interest. Table 2 shows the top 30 genes for which expression levels were higher in the proximal DA than in the distal DA. Table 3 shows the top 30 genes for which expression levels were lower in the proximal DA than in the distal DA. To profile the genes, which have distinct expression levels between the proximal DA and the distal DA, we performed gene ontology analysis using the DAVID knowledgebase. We found that the genes in the melanogenesis and tyrosine metabolism pathways were significantly enriched in the proximal DA compared to the distal DA (Table 4). It should be noted that all of the genes shown in the tyrosine metabolism pathway overlapped with the genes in the melanogenesis pathway.

**Table 2:** Top 30 genes with high proximal DA/distal DA ratio.

Gene name	Gene symbol	Fold change (proDA/disDA)	NCBI ref seq	Probe ID	gene ID
tyrosinase (oculocutaneous albinism IA)	*tyr*	1.63	NM_204160	15408323	373971
hematopoietic prostaglandin D synthase	*hpgds*	1.53	NM_205011	15509514	395863
glycoprotein (transmembrane) nmb	*gpnmb*	1.49		15450041	428431
lysosomal-associated membrane protein 3	*lamp3 *	1.45	NM_001146132	15546981	424961
tyrosinase-related protein 1	*tyrp1 *	1.44	NM_205045	15558998	395913
cytochrome P-450 2C45	*cyp2c45*	1.41		15526246	414833
mucin protein	*muc*	1.4	AJ487010	15520261	404528
dopachrome tautomerase (dopachrome delta-isomerase, tyrosine-related protein 2)	*dct*	1.36	NM_204935	15394509	395775
epithelial cell adhesion molecule	*epcam*	1.35	NM_001012564	15485994	421292
surfactant, pulmonary-associated protein A1	*sftpa1*	1.34	NM_204606	15528597	395308
proteoglycan 4	*prg4*	1.33		15542771	100859371
chordin-like 1	*chrdl1*	1.32	NM_204171	15501860	373985
EMI domain containing 2	*emid2*	1.32	CR387598	15444301	417504
uroplakin 1B	*upk1b*	1.31		15402890	418345
membrane metallo-endopeptidase	*mme *	1.31	NM_001004412	15550074	425031
transcription factor AP-2 beta (activating enhancer binding protein 2 beta)	*tfap2b*	1.3	NM_204895	15500239	395713
coagulation factor III (thromboplastin, tissue factor)	*f3*	1.29	XM_426640	15539617	429084
mucin 22	*muc22 *	1.29		15520233	
thymosin, beta 10	*tmsb10*	1.29	XM_001234311	15425780	770993
myelin basic protein	*mbp*	1.28	NM_205280	15452918	396217
GDNF family receptor alpha 1	*gfra1*	1.28	NM_205102	15530884	395994
tenascin-R-like	*tnxb*	1.28		15443898	427816
lung lectin	*ll*	1.28	NM_001039166	15528591	423630
cytokine-like 1	*cytl1*	1.27	BX931297	15512352	422849
protocadherin-related 15	*pcdh15*	1.27	NM_001044654	15525136	423644
interleukin 1 receptor-like 1	*il1rl1*	1.26	NM_204275	15393753	374136
melan-A	*mlana*	1.26	BX931525	15558939	769648
cholinergic receptor, muscarinic 2	*chrm2*	1.26	NM_001030765	15401066	418126
MAM domain containing 2	*mamdc2*	1.26	BX935251	15559233	427247

**Table 3:** Top 30 genes with low proximal DA/distal DA ratio.

Gene name	Gene symbol	Fold change (proDA/disDA)	NCBI ref seq	Probe ID	Gene ID
guanylate cyclase 1, soluble, alpha 3	*gucy1a3*	0.73	BX933178	15502379	422407
protein tyrosine phosphatase, receptor type, O	*ptpro*	0.77	NM_204122	15389071	373911
heparanase 2	*hpse2*	0.78	XM_421704	15526875	423834
urocortin 3	*ucn3*	0.78	BX930520	15397182	769274
SPARC related modular calcium binding 1	*smoc1*	0.79	XM_001231760	15521884	768688
glutamate receptor, ionotropic, N-methyl-D-aspartate 3A	*grin3a*	0.82		15560556	769001
angiotensin I converting enzyme (peptidyl-dipeptidase A) 1	*ace*	0.82	NM_001167732	15480996	419953
collagen, type XXIII, alpha 1	*col23a1*	0.82		15424012	425481
growth differentiation factor 5	*gdf5*	0.83	NM_204338	15464465	374249
ADAM metallopeptidase domain 22	*adam22*	0.83	NM_001145228	15449411	420537
signal peptide, CUB domain, EGF-like 1	*scube1*	0.83	XM_416453	15401722	418228
hepatocyte growth factor (hepapoietin A; scatter factor)	*hgf*	0.84	NM_001030370	15384844	395941
nebulette	*nebl*	0.84	NM_204488	15449124	395148
collagen, type XV, alpha 1	*col15a1*	0.84		15451458	420803
Golgi integral membrane protein 4-like	*loc419409*	0.84		15468540	419409
opioid receptor, mu 1	*oprm1*	0.85		15489262	421644
overexpressed in colon carcinoma 1 protein homolog	*occ-1*	0.85	NM_001145200	15400640	771624
angiopoietin 1	*angpt1*	0.85	NM_001199447	15463268	395129
heparan sulfate (glucosamine) 3-O-sulfotransferase 1	*hs3st1*	0.85	BX934256	15505973	422840
erythrocyte membrane protein band 4.1-like 3	*epb41l3*	0.85		15453301	421055
C1q and tumor necrosis factor related protein 3	*c1qtnf3*	0.85	BX933888	15561814	427430
neuropeptide Y	*npy*	0.85	NM_205473	15450094	396464
carboxypeptidase X (M14 family), member 2	*cpxm2*	0.85		15531136	423951
feather keratin Cos1-1/Cos1-3/Cos2-1-like	*loc431350*	0.85		15480612	431350
FERM and PDZ domain containing 4	*frmpd4*	0.85		15405134	418640
NEL-like 2 (chicken)	*nell2*	0.86	NM_001030740	15398562	417799
sema domain, immunoglobulin domain (Ig), short basic domain, secreted, (semaphorin) 3A	*sema3a*	0.86	NM_204977	15384739	395825
odz, odd Oz/ten-m homolog 1 (Drosophila)	*odz1*	0.86	NM_204862	15501976	395668
microRNA mir-103-2	*mir103-2*	0.86	NR_031437	15513017	777817
1-phosphatidylinositol-4,5-bisphosphate phosphodiesterase epsilon-1-like	*loc425224*	0.86		15553001	425224

**Table 4:** Gene ontology analysis for genes with high proximal DA/distal DA ratio.

Category	Term	Count	%	P Value	Genes	List Total	Pop Hits	Pop Total	Fold Enrichment	Bonferroni	Benjamini	FDR
KEGG_PATHWAY	gga04916: Melanogenesis	5	6.5	0.0009	*tyr*, *wnt11*, *ednrb2*, *dct*, *tyrp1*	23	89	4353	10.6	0.025	0.025	0.75
KEGG_PATHWAY	gga00350: Tyrosin metabolism	3	3.9	0.01	*tyr*, *dct*, *tyrp1*	23	31	4353	18.3	0.25	0.14	8.4

Since the DA is known to be highly oxygen-sensitive, hypoxia-dependent genes are of great interest. Therefore, we analyzed whether the genes that are expected to involve in hypoxia-dependent regulation are predominantly expressed in the proximal DA. Among 206 genes categorized as “response to hypoxia” in GO term, 155 gene probes were found in the chick DNA microarray. Among 155 probes, 4 genes (*ada*, *chrna4*, *kdr*, *rora*) were identified from the top 0.5% in the ratio of signal intensities of the proximal DA to the distal DA or proximal DA to the descending aorta (Table 5). Among 72 genes categorized as “HIF-1 signaling pathway” in KEGG pathway, 65 gene probes were found in the chick DNA microarray. Among 65 probes, *angpt1* was solely identified from the top 0.5% in the ratio of signal intensities of the proximal DA to the distal DA (Table 5).

**Table 5:** Hypoxia-related genes from the top 0.5% in the ratio of signal intensities of the proximal DA to the distal DA.

Gene name	Gene symbol	Fold change (proDA/disDA)	Fold change (proDA/aorta)	Fold change (disDA/aorta)	NCBI ref seq	Probe ID	gene ID
adenosine deaminase	*Ada*	0.896	0.813	0.907	NM_001006290	15464840	419194
cholinergic receptor, nicotinic, alpha 4	*Chrna*	0.888	0.902	1.016	NM_204814	15467262	395606
kinase insert domain receptor (a type III receptor tyrosine kinase)	*Kdr*	1.011	1.339	1.324	NM_001004368	15505176	395323
RAR-related orphan receptor A	*Rora*	0.887	0.904	1.019	NM_001289887	15409684	415377
angiopoietin 1	*Angpt1*	0.847	1.01	1.192	NM_001199447	15463268	395129

### PCR analyses confirmed distinct transcriptional profiles of the chick DA

To verify the results of the microarray, we performed quantitative RT-PCR for collagen type VIII alpha 1, *col8a1*, tenascin C, *tnc*, MAM domain containing 2, *mamdc2*, heparanase 2, *hpse2*, and family with sequence similarity 19 member A2, *fam19a2*. We found that *col8a1* (Fig 2A), *tnc* (Fig 2B), and *mamdc2* (Fig 2C) were highly expressed in the proximal DA compared to the distal DA and the aorta at the IP stage. On the other hand, the expression levels of *hpse2* (Fig 2D) and *fam19a2* (Fig 2E) were significantly lower in the proximal DA than those in the distal DA and the aorta at the IP stage. These data supported the results of the DNA microarray. Furthermore, we found that the expression levels of *col8a1* were significantly higher in the proximal DA than those in the aorta at the ED16, ED19, EP, and AB stages. The expression levels of *tnc* were significantly higher in the proximal DA than those in the distal DA and the aorta at the ED19, IP, and EP stages and rapidly declined at the AB stage. The expression levels of *mamdc2* were significantly upregulated in the proximal DA from ED19 and were significantly higher in the proximal DA than those in the aorta after ED19. Interestingly, the difference of the expression levels of *hpse2* and *fam19a2* also became apparent from ED19.

Next, we examined the expression levels of genes related to melanogenesis and/or tyrosine metabolism that were upregulated in the proximal DA at IP. We found that the expression of *dct* (Fig 3A) and *ednrb2* (Fig 3B) and *tyr* (Fig 3C) in the proximal DA was fairly higher than that in the distal DA at the IP stage, because the expression of these genes in the distal DA was below the detection limit. The expression peaks of *dct* and *ednrb2* in the proximal DA occurred at the IP stage, whereas that of *tyr* occurred at ED19. We hardly detected the expression of *ednrb2*, *dct*, and *tyr* in the aorta at any of the stages. The expression levels of *tyrp1* were significantly higher in the proximal DA than those in the distal DA and the aorta at the IP stage (Fig 3D). The expression levels of *wnt11* were significantly higher in the proximal DA than those in the distal DA and the aorta at the ED16 stage (Fig 3E).

## Discussion

Our microarray analyses uncovered that the gene expression profiles between the proximal and distal ductus arteriosi differed, although they were much closer than that of the aorta during chick development. To our knowledge, this is the first study examining the transcription profiles of the chicken DA. Importantly, the proximal DA is morphologically and functionally distinct from the distal DA, which more resembles the descending aorta [1, 2, 6, 7, 20]. However, our DNA microarray data indicated that gene expression profiles between the proximal and distal ductus arteriosi were much closer than that of the aorta. It is known that the smooth muscle cells of DA are derived entirely from cardiac neural crest cells, whereas smooth muscle cells of the aorta are primarily derived from mesenchymal cells [21–23]. This may account for the similarity of transcriptional profiles between the proximal and distal ductus arteriosi when compared to that of the descending aorta. Among 30 genes, 17 genes are more highly expressed in the proximal and distal DA than in the aorta. Among them, the transcription factor *tfap2b* is of particular interest because this gene expression apparently showed a gradient change from the proximal DA to the aorta (expression in the proximal DA > that in the distal DA > that in the aorta). It has been known that mutations in *tfap2b* cause Char syndrome, a familial form of patent ductus arteriosus in humans [24–26] and that mutations or deficiency in *tfap2b* cause patent ductus arteriosus in mice [27, 28]. These data indicate that *tfap2b* is very well conserved among species and plays a critical role in the development of the DA. In addition to *tfap2b*, *tnc* [29], *des* [11, 13, 15, 29], *foxf1* [14], *ankrd1* [11], *mgp* [13], *atp2a3* [10], and *fhl2* [15, 30] are also reported to have a higher expression pattern in chickens and other species. Yarboro et al. has recently reported the transcriptional profiling of human DA and compared to the published rodent data [8]. We then found that 11 genes are common to both studies when our data were compared with Yarboro's human data (S5 Table). Most of the genes show the similar dominant expression patterns between chick and human ductus arteriosi. Among them, we also confirmed the expression levels of *col8a1*, *mamdc2*, *hpse2*, and *fam19a2* by RT-PCR analyses during development. These proximal DA-dominant genes of which expression is conserved well in many species may play an important role in the regulation of DA development, although the specific function of these genes in the DA has not yet been determined. Interestingly, the transcription factor *foxf1* is known to bind to serum response factor and myocardin to regulate gene transcription in visceral smooth muscle cells [31]. In addition, *foxf1* is a candidate gene for familial patent ductus arteriosus [32]. Moreover, homozygous deletion of the *foxc1* gene is known to cause failure of DA closure in mice [33]. Therefore, further investigation would be needed to identify the role of forkhead box genes in DA development.

We assume that the genes distinctly expressed between the proximal and distal ductus arteriosi should be involved in generating the morphological and functional differences between the proximal DA and the distal DA. Interestingly, gene ontology analysis revealed that the differentially expressed transcripts were involved in melanogenesis and tyrosine metabolism of the chicken DA. In mice, tyrosinase promoter is transiently active from day 9 of gestation in common precursors of melanocytes and smooth muscle cells in the DA [34]. Furthermore, a certain population of these precursor cells should be differentiated from melanoblasts to smooth muscle cells in the DA, which is important for proper closure of the DA after birth. Therefore, our results also confirmed that tyrosinase and the related genes play an important role in proper differentiation of neural crest-derived cells in the process of vascular remodeling in the DA. On the other hand, we found that only a small number of genes related with hypoxia-dependent regulation showed a different expression pattern between proximal DA and the aorta at IP. This may be due to the stage we examined. Because a previous study has demonstrated that hypoxic inducible factor 2a, *hif2a*, plays an important role in DA development in mice [35], examining the hypoxia-related genes at other developmental stages should be required in chick in our future study.

The role of most of the genes in Table 2 and Table 3 has not been uncovered in the DA. Because, despite the morphological differences, there are many similarities in the molecular mechanisms of DA closure between chickens and mammals [1, 2, 6, 7, 20], it would be intriguing to investigate the function of the genes listed in Table 2 and Table 3 to discover a new molecule involved in DA closure in humans.

## Conclusions

### Conclusion

The present DNA microarray analysis newly found genes that were distinctly expressed in the chicken proximal DA. Furthermore, cluster analysis found that the expression pattern of the proximal DA was closer to that of the distal DA than that of the aorta, although the proximal and distal ductus arteriosi in chicks have distinct characteristics such as oxygen responsiveness. The transcription profiles of the chick DA would provide a new insight in investigating the mechanism of DA closure.

### Limitation

In the present study, we only compared the gene profiles of chick DA at the IP stage. The main aim of the present study is to examine comprehensive transcriptional profiles of chick DA because no study has been conducted to examine a transcriptional profile of the DA of oviparous animals. We thought that the DA at the IP stage is rapidly differentiated and that the functional and morphological differences are becoming apparent. Therefore, we chose the IP stage for our first investigation. It would be interesting, however, to compare the transcriptional profiles of the chicken DA during development.

## Supporting information

S1 Table Top 30 genes with high proximal DA/aorta ratio. (PDF)

S2 Table Top 30 genes with high distal DA/aorta ratio. (PDF)

S3 Table Top 30 genes with low proximal DA/aorta ratio. (PDF)

S4 Table Top 30 genes with low distal DA/aorta ratio. (PDF)

S5 Table Eleven common genes in chick and human ductus arteriosi. (PDF)
