# Supplementary material for: Transcriptional profiles in the chicken ductus arteriosus during hatching
Source: PLoS One. 2019 Mar 21;14(3):e0214139. doi: 10.1371/journal.pone.0214139 (PMC6428269; doi:10.1371/journal.pone.0214139)
Supplement: S1 Table — (PDF) [file pone.0214139.s001.pdf]

S1 Table. Top 30 genes with high proximal DA/aorta ratio.

Gene name	Gene symbol	Fold Change (proDA/aorta)	NCBI ref seq	Probe ID	gene ID
tenascin C	*tnc*	2.15	NM_205456	15439065	396440
collagen, type VIII, alpha 1	*col8a1*	1.98		15403120	418378
family with sequence similarity 132, member A	*fam132a*	1.82	XM_417583	15468686	419422
transcription factor AP-2 beta	*tfap2b*	1.70	XM_417583	15500239	395713
family with sequence similarity 13, member C	*fam13c*	1.69		15524862	423602
regulator of G-protein signaling 16	*rgs16*	1.68	BX931668	15538900	424409
serine peptidase inhibitor, Kazal type 5	*spink5*	1.59	NM_001030612	15423382	416235
desmin	*des*	1.58		15536995	395906
calcium channel, voltage-dependent, T type, alpha 1H subunit	*cacna1h*	1.57		15429785	416526
hematopoietic prostaglandin D synthase	*hpgds*	1.56	NM_205011	15509514	395863
G protein-coupled receptor 20	*gpr20*	1.56		15464028	428381
solute carrier family 38, member 4	*slc38a4*	1.55	NM_001199549	15398648	417809
tenascin C	*tnc*	1.54	X73833	15439092	396440
glycoprotein (transmembrane) nmb	*gpnmb*	1.54		15450041	428431

clone ChEST566j15		1.54	CR385930	15517930	
tyrosinase (oculocutaneous albinism IA)	*tyr*	1.54	NM_204160	15408323	373971
phytanoyl-CoA 2-hydroxylase interacting protein-like	*phyhipl*	1.53	NM_001199504	15528343	423603
c-fos induced growth factor (vascular endothelial growth factor D)	*figf*	1.50	NM_204568	15393053	395255
GDNF family receptor alpha 1	*gfra1*	1.48	NM_205102	15530884	395994
forkhead box F1	*foxf1*	1.48	AB028627	15417901	395624
tyrosinase-related protein 1	*tyrp1*	1.48	NM_205045	15558998	395913
ankyrin repeat domain 1 (cardiac muscle)	*ankrd1*	1.47	NM_204405	15529946	378926
matrix metallopeptidase 11 (stromelysin 3)	*mmp11*	1.47		15432843	769489
MAM domain containing 2	*mamdc2*	1.47	BX935251	15559233	427247
transgelin	*tagln*	1.45		15475181	396490
matrix Gla protein	*mgp*	1.45	NM_205044	15387274	395912
cytokine-like 1	*cytl1*	1.45	BX931297	15512352	422849
potassium large conductance calcium-activated channel, subfamily M, beta member 2	*kcnmb2*	1.45	BX950825	15549669	770907
dopachrome tautomerase (dopachrome delta-isomerase, tyrosine-related protein 2)	*dct*	1.44	NM_204935	15394509	395775
