# Supplementary material for: Transcriptional profiles in the chicken ductus arteriosus during hatching
Source: PLoS One. 2019 Mar 21;14(3):e0214139. doi: 10.1371/journal.pone.0214139 (PMC6428269; doi:10.1371/journal.pone.0214139)
Supplement: S2 Table — (PDF) [file pone.0214139.s002.pdf]

S2 Table. Top 30 genes with high distal DA/aorta ratio.

Gene name	Gene symbol	Fold Change (disDA/aorta)	NCBI ref seq	Probe ID	gene ID
tenascin C	*tnc*	1.82	NM_205456	15439065	396440
collagen, type VIII, alpha 1	*col8a1*	1.69		15403120	418378
family with sequence similarity 132, member A	*fam132a*	1.59	XM_417583	15468686	419422
family with sequence similarity 13, member C	*fam13c*	1.59		15524862	423602
G protein-coupled receptor 20	*gpr20*	1.45		15464028	428381
phytanoyl-CoA 2-hydroxylase interacting protein-like	*phyhipl*	1.44	NM_001199504	15528343	423603
desmin	*des*	1.41		15536995	395906
calcium channel, voltage-dependent, T type, alpha 1H subunit	*cacna1h*	1.40		15429785	416526
clone ChEST566j15		1.38		15517930	
matrix Gla protein	*mgp*	1.38	NM_205044	15387274	395912
tenascin	*tnc*	1.37	X73833	15439092	396440
melanocortin 5 receptor	*mc5r*	1.36	NM_001031015	15461279	421047
regulator of G-protein signaling 16	*rgs16*	1.35		15538900	424409
G protein-coupled receptor 126	*gpr126*	1.35	NM_001031071	15497320	421673

ATPase, Ca++ transporting, ubiquitous	*atp2a3*	1.33	NM_204891	15444194	395707
serine peptidase inhibitor, Kazal type 5	*spink5*	1.33	NM_001030612	15423382	416235
kinase insert domain receptor (a type III receptor tyrosine kinase)	*kdr*	1.32	NM_001004368	15505176	395323
transgelin	*tagln*	1.32		15475181	396490
family with sequence similarity 20, member C	*fam20c*	1.32		15426628	416445
leucine-rich repeat containing G protein-coupled receptor 5	*lgr5*	1.32		15386401	427867
solute carrier family 38, member 4	*slc38a4*	1.32	NM_001199549	15398648	417809
G protein-coupled receptor 116	*gpr116*	1.31		15500349	422061
transcription factor AP-2 beta (activating enhancer binding protein 2 beta)	*tfap2b*	1.31	NM_204895	15500239	395713
von Willebrand factor	*vwf*	1.31		15401973	419031
microRNA mir-1645	*mir1645*	1.29	NR_035136	15398276	100316010
c-fos induced growth factor (vascular endothelial growth factor D)	*figf*	1.29	NM_204568	15393053	395255
nidogen 1	*nid1*	1.28		15488286	395531
four and a half LIM domains 2	*fhl2*	1.27	BX933997	15405749	418726
microRNA mir-27b	*mir27b*	1.27	NR_031497	15559626	777878
