# Supplementary material for: Transcriptional profiles in the chicken ductus arteriosus during hatching
Source: PLoS One. 2019 Mar 21;14(3):e0214139. doi: 10.1371/journal.pone.0214139 (PMC6428269; doi:10.1371/journal.pone.0214139)
Supplement: S3 Table — (PDF) [file pone.0214139.s003.pdf]

S3 Table. Top 30 genes with low proximal DA/aorta ratio.

Gene name	Gene symbol	Fold change (proDA/aorta)	NCBI ref seq	Probe ID	gene ID
microRNA 1329	*mir1329*	0.64	NR_035009	15492062	100315694
solute carrier family 1 (glial high affinity glutamate transporter), member 3	*slc1a3*	0.67		15557705	395443
guanylate cyclase 1 soluble subunit alpha	*gucy1a3*	0.67	BX933178	15502379	422407
angiotensin I converting enzyme	*ace*	0.68	NM_001167732	15480996	419953
spermatogenesis associated 13	*spata13*	0.69	CR389315	15395669	418940
opioid receptor, mu 1	*oprm1*	0.69		15489262	421644
spermatogenesis associated 13	*spata13*	0.70		15395671	418940
clone 840 GGN101 snoRNA		0.70	EU240316	15536100	
family with sequence similarity 46, member C	*fam46*	0.70	NM_001007972	15402678	426544
GLIS family zinc finger 1	*glis1*	0.71		15544262	424652
heparanase 2	*hpse2*	0.71	XM_421704	15526875	423834
papilin, proteoglycan-like sulfated glycoprotein	*papln*	0.72		15521735	42887
protein tyrosine phosphatase, receptor type, O	*ptpro*	0.72	NM_204122	15389071	373911
KIAA1644	*kiaa1644*	0.74		15401776	100857276

adenylate cyclase activating polypeptide 1 (pituitary) receptor type I	*adcyap1r1*	0.74	NM_001098606	15448093	420386
alpha-1,4-N-acetylglucosaminyltransferase	*a4gnt*	0.74	XM_426692	15545524	429136
hepatocyte growth factor (hepapoietin A; scatter factor)	*hgf*	0.74	NM_001030370	15384844	395941
CUB and Sushi multiple domains 1	*csmd1*	0.74		15491766	421899
gamma-aminobutyric acid (GABA) A receptor, beta 2	*gabrb2*	0.75	X72806	15422949	414890
collagen, type XV, alpha 1	*col15a1*	0.75		15451458	420803
protein phosphatase 4, regulatory subunit 4	*ppp4r4*	0.75	XM_421339	15517524	423430
single-minded homolog 2 (Drosophila)	*sim2*	0.75	XM_416724	15392089	418515
neuropeptide Y	*npy*	0.76	NM_205473	15450094	396464
family with sequence similarity 19 (chemokine (C-C motif)-like), member A2	*fam19a2*	0.76		15398714	771745
Ig heavy chain V-III region VH26-like	*loc430014*	0.76		15554626	430014
collagen, type XXI, alpha 1	*col21a1*	0.76		15491621	421885
family with sequence similarity 110, member B	*fam110b*	0.76	XM_419215	15453999	421134
ATPase, H+ transporting, lysosomal 42kDa, V1 subunit C2	*atp6v1c2*	0.76	XM_419951	15492047	421939
collagen, type XXIII, alpha 1	*col23a1*	0.76		15424012	425481
leucine rich repeat containing 8 family, member B	*lrrc8b*	0.77		15543240	424516
