# Supplementary material for: Transcriptional profiles in the chicken ductus arteriosus during hatching
Source: PLoS One. 2019 Mar 21;14(3):e0214139. doi: 10.1371/journal.pone.0214139 (PMC6428269; doi:10.1371/journal.pone.0214139)
Supplement: S4 Table — (PDF) [file pone.0214139.s004.pdf]

S4 Table. Top 30 genes with low distal DA/aorta ratio.

Gene name	Gene symbol	Fold change (disDA/aorta)	NCBI ref seq	Probe ID	gene ID
microRNA mir-1329	*mir1329*	0.68	NR_035009	15492062	100315694
GGN101 snoRNA		0.69	EU240316	15536100	
GLIS family zinc finger 1	*glis1*	0.70	XM_015291201	15544262	424652
solute carrier family 1 (glial high affinity glutamate transporter), member 3	*slc1a3*	0.73		15557705	395443
snail homolog 2 (Drosophila)	*snai2*	0.74	CR407272	15551818	432368
protein phosphatase 4, regulatory subunit 4	*ppp4r4*	0.74	XM_421339	15517524	423430
ATPase, H+ transporting, lysosomal 42kDa, V1 subunit C	*atp6v1c2*	0.74	XM_419951	15492047	42193
beta-defensin 12	*gal12*	0.76	NM_00100160	15500179	414339
spermatogenesis associated 13	*spata13*	0.76		15395671	418940
adenylate cyclase activating polypeptide 1 (pituitary) receptor type I	*adcyap1r1*	0.77	NM_001098606	15448093	420386
spermatogenesis associated 13	*spata13*	0.77	CR389315	15395669	418940
transmembrane protein 71	*tmem71*	0.78	XM_025148183	15463797	101749956
snail homolog 2 (Drosophila)	*snai2*	0.78	CR407272	15462084	432368
KIAA1644	*kiaa1644*	0.78		15401776	100857276

CUB and Sushi multiple domains 1	*csmd1*	0.78		15491766	421899
periostin, osteoblast specific facto	*postn*	0.78	NM_001030541	15395306	395429
BARX homeobox 2	*barx2*	0.79	NM_204896	15474796	395714
papilin, proteoglycan-like sulfated glycoprotei	*papln*	0.79		15521735	42887
clone 583 noncoding RNA GGN52 gene		0.79	EU240268	15539164	
alpha-1,4-N-acetylglucosaminyltransferase	*a4gnt*	0.80	XM_42669	15545524	429136
clone 753 GGN112 snoRN		0.80	EU240326	15563349	
single-minded homolog 2 (Drosophila)	*sim2*	0.80	XM_416724	15392089	418515
collagen, type XXI, alpha 1	*col21a1*	0.80		15491621	421885
unc-13 homolog B (C. elegans)	*unc13*	0.80	XR_140354	15557363	
leucine rich repeat containing 8 family, member B	*lrrc8b*	0.80		15543240	424516
IQ motif containing GTPase activating protein 2	*iqgap2*	0.81		15562712	427211
potassium voltage-gated channel, KQT-like subfamily, member	*kcnq3*	0.81		15463768	420325
family with sequence similarity 19 (chemokine (C-C motif)-like), member	*fam19a2*	0.81		15398714	771745
LY6/PLAUR domain containin	*lypd6*	0.81	XM_001234905	15534569	771649
3617 GGN113 snRNA		0.81	EU240327	15552097	
