# Supplementary material for: Transcriptional profiles in the chicken ductus arteriosus during hatching
Source: PLoS One. 2019 Mar 21;14(3):e0214139. doi: 10.1371/journal.pone.0214139 (PMC6428269; doi:10.1371/journal.pone.0214139)
Supplement: S5 Table — (PDF) [file pone.0214139.s005.pdf]

S5 Table. Eleven common genes in chick and human ductus arteriosi.

Gene symbol	Chicken microarray	literature	
		comparison	Species
mme	Proximal DA>distal DA	DA>aorta	Human
Chrm2	Proximal DA>distal DA	DA>aorta	Human
Mamdc2	Proximal DA>distal DA<br>Proximal DA>aorta	DA>aorta	Human
Scube1	Distal DA>proximal DA	DA>aorta	Human
C1qtnf3	Distal DA>proximal DA	DA>aorta	Human
Col8a1	Distal DA>aorta	DA>aorta	Human
Hpse2	Distal DA>proximal DA<br>Aorta>proximal DA	Aorta>DA	Human
Adam22	Distal DA>proximal DA	Aorta>DA	Human
Gabrb2	Aorta>proximal DA	Aorta>DA	Human
Fam19a2	Aorta>proximal DA<br>Aorta>distal DA	Aorta>DA	Human
postn	Aorta>distal DA	Aorta>DA	Human
